# Urine-derived mesenchymal stem cells-derived exosomes enhances survival and proliferation of aging retinal ganglion cells

**DOI:** 10.1186/s12860-023-00467-4

**Published:** 2023-03-07

**Authors:** Qi-Qin Dan, Li Chen, Lan-Lan shi, Xiu Zhou, Ting-Hua Wang, Hua Liu

**Affiliations:** 1 Present Address: Institute of Neurological Disease, West China Hospital, Sichuan University, No. 88 Keyuan South Road, Chengdu, 610041 China; 2 Department of Human Anatomy and Tissue Embryology, Kunming Medical University, Kunming, 650500 China; 3 Laboratory Animal Department, Institute of Neuroscience, Kunming Medical University, Kunming, 650500 China; 4 Department of Anatomy, Institute of Eyes, Jinzhou Medical University, No.40, Section 3, Songpo Road, Linghe District, JinzhouLiaoning, 121001 China

**Keywords:** Retinal ganglion cells, Urine-derived stem cells-derived exosomes, Aging-related RGCs function loss, Cell viability and proliferation

## Abstract

**Objectives:**

This study was designed to investigate to test the effect of exosomes from urine-derived mesenchymal stem cells (USCs) on the survival and viability of aging retinal ganglion cells (RGCs), and explored the preliminary related mechanisms.

**Methods:**

Primary USCs were cultured and identified by immunofluorescence staining. Aging RGCs models were established by D-galactose treatment and identified by β-Galactosidase staining. After treatment with USCs conditioned medium (with USCs removal), flow cytometry was performed to examine the apoptosis and cell cycle of RGCs. Cell viability of RGCs was detected by Cell-counting Kit 8 (CCK8) assay. Moreover, gene sequencing and bioinformatics analysis were applied to analyze the genetic variation after medium treatment in RGCs along with the biological functions of differentially expressed genes (DEGs).

**Results:**

The number of apoptotic aging RGCs was significantly reduced in USCs medium-treated RGCs. Besides, USCs-derived exosomes exert significant promotion on the cell viability and proliferation of aging RGCs. Further, sequencing data analyzed and identified DEGs expressed in aging RGCs and aging RGCs treated with USCs conditioned medium. The sequencing outcomes demonstrated 117 upregulated genes and 186 downregulated genes in normal RGCs group vs aging RGCs group, 137 upregulated ones and 517 downregulated ones in aging RGCs group vs aging RGCs + USCs medium group. These DEGs involves in numerous positive molecular activities to promote the recovery of RGCs function.

**Conclusions:**

Collectively, the therapeutic potentials of USCs-derived exosomes include suppression on cell apoptosis, enhancement on cell viability and proliferation of aging RGCs. The underlying mechanism involves multiple genetic variation and changes of transduction signaling pathways.

## Introduction

Retinal ganglion cells (RGCs) are nerve cells located in the final segment of retina. RGCs axons are optic nerve fibers in the eyeball, which are distributed on the surface of omentum and collected in the papilla of optic tract (optic nerve) [1, 2]. After coming out of the eyeball, they pass through the intersection of optic tract and end in the lateral geniculate body. In the retina, photoreceptors transmit the visual information to interneurons which process and pass on the information to RGCs, axons of which then travel through the optic nerve, informing the rest of the brain of all it will ever know about the visual world [3, 4]. It has been reported that there are appropriately 30 types of RGCs, and distinct RGC types exhibit different abilities to survive diseases like glaucoma or surgical trauma like axotomy [5]. Considerable efforts have been dedicated in previous studies to identify the cascade of structural and functional alterations in RGCs that eventually lead to their death [6]. Previous studies demonstrated that RGCs function is compromised within a short period after intraocular pressure (IOP) increase [7, 8], followed by notable morphological variation in dendrites. The subsequently progressive loss and death of RGCs result in vision loss. Accumulating evidences from experimental and clinical studies suggest that RGCs are capable of recovering themselves after periods of functional loss at early onset of glaucoma, however, the abilities to recover functions may be negatively impacted with aging [9]. With aging, the function loss of RGCs was aggravated gradually. It’s of vital importance to find the effective therapy to prevent aging-related RGCs function loss and boost functional recovery of RGCs in the progression of aging.

Among a variety of approaches applied to ameliorate vision loss, lowering IOP is the most widely used [10]. Nevertheless, it’s quite tough work to hinder the aging process of RGCs as human get older. Stem cells are a kind of pluripotent cells with strong self-replication abilities which enable them differentiate into a variety of functional cells and have the potential function of regenerating various tissues and organs. Currently, it’s quite familiar to us with various types of stem cells, such as neural stem cells, embryonic stem cells, bone marrow mesenchymal stem cells, umbilical cord mesenchymal stem cells, etc., which have been implicated in a great many disorders and diseases. Urine-derived mesenchymal stem cells (USCs) can be obtained from patients' urine samples in a non-invasive way, and these cells in urine can differentiate into bladder-like cells, such as smooth muscle and urinary tract epithelial cell lines, even embryonic stem cells [11, 12]. At the same time, USCs are revealed to be involved in the formation of bone, soft bone, skeletal muscle and other tissues [13]. The pluripotency of USCs reveals potency that they might be used in the treatment of numerous diseases. Such functions are mediated through the secretion of particles from USCs and resident cells known as extracellular vesicles (EVs) within a microenvironment. EVs are a heterogeneous population of membranous vesicles released from cells that transfer many types of active biomolecules to recipient cells and induce physiologic and phenotypic alterations in the tissue environment [14]. Exosomes, a subtype of EVs mediate intercellular communication, target cells inducing cellular development through different signaling pathways. EVs like exosomes originating from the endosomal pathway carry different types of biomolecules such as nucleic acids, proteins, and lipids; participate in cell-to-cell communication [15]. Targeting exosomes represents novel approaches for the treatment of cancers through using them as cell-free therapy and drug-delivery system and inhibiting their biogenesis and distribution [16]. Mesenchymal stem cells (MSCs) derived exosomes have great potentials to improve ageing and age-related diseases [17]. Exosomes-therapy may serve as a new approach to combat aging.

In this study, we investigated the effect of USCs-derived exosomes on preventing the functional loss of aging RGCs and preliminarily analyzed the underlying mechanisms. Our finding revealed that treatment with the conditioned USCs medium reversed the aging progression of RGCs cells, which provide novel insights into the prevention and treatment of vision disorders.

## Materials and methods

### Cell culture

RGCs used in this study were purchased from Qingqi Biotechnology Development Co., Ltd. (Shanghai, China). RGCs were cultured with high glucose medium containing 10% inactivated fetal bovine serum (FBS) in an incubator with 5% CO_2_ at 37 ℃. When RGCs reached 80%—90% confluence and grew well, they were digested with trypsin and subcultured.

Urine was collected from healthy adult females, and USCs were separated and cultured in special medium of urine stem cell which were purchased from Xihan Biotechnology Co., Ltd. (Shanghai, China), incubated with 5% CO_2_ at 37℃. When the cell confluence reached about 85%, they were generally subcultured every 3–5 days. If the cells grew well with 85% cell confluence, it is recommended to subculture at ratio of 1:3. Otherwise, the cells with lower density can be subcultured at ratio of 1:2. There’re some precautions in the process of operation. Before passage, a warm bath at 37 ℃ is necessary for trypsin, and digestion should be carried out in a 37 ℃ incubator. The duration of digestion shall be controlled within about 1 min to avoid affecting the cell state. The third-generation cells were used in the following experiments.

### Identification of USCs by immunofluorescence (IF) staining

USCs, primarily cultured in our laboratory, were identified by flow cytometry in Yunnan Key Laboratory of Stem Cell Translation. The biological characteristics of USCs resemble to those of MSCs, and it has been shown that USCs highly express four markers, CD44, CD73, CD90 and CD105. Identification of MSCs was performed as previously described [18, 19]. The cells were prepared into single cell suspension, 5 μl of CD44 and CD90 antibodies were added and incubated for 30 min at room temperature in dark. Afterwards, they were washed twice with phosphate buffer (PBS, pH 7.2) and centrifuged at 1,500 r/min for 5 min. Then, the 200 μl of PBS was added, and the cells were reconstituted into cell suspension for detection on florescence intensity of cells by flow cytometry [20].

### Cell counting kit (CCK8) assay

RGCs in logarithmic growth stage were inoculated in 96 well plate (200 μL/well) at a density of 1.6 × 10^5^/ml. There were blank group, normal group and aging groups, with 6 replicated wells in each group. The blank group did not contain cells. The cells in the normal group were cultured in complete medium composed of high glucose medium, 10% FBS, 1% penicillin–streptomycin double antibodies. The cells in the aging group were cultured in complete culture medium with D-galactose at concentrations of 5, 10, 20, 30 and 40 mg/ml for 24 h in an incubator with 5% CO_2_ at 37 ℃, and then the complete culture medium was changed for another 24-h incubation. Subsequently, 10 μL CCK-8 solution per 100 μL medium was added into each well. After continuous culture for half hour, the absorbance value (A) of each well at the wavelength of 450 nm was detected using a microplate reader and the cell survival rate was calculate as: cell survival rate (%) = (A value of test group—A value of blank group) / (A value of normal group—A value of blank group) × 100%. The test was repeated 3 times.

### β-Galactosidase staining of aging cells

RGCs in logarithmic growth stage were inoculated into 6-well plates at a density of 1 × 10^4^ cells. The cells were arranged into normal group, aging group and USCs conditioned medium (with USCs removal) group. After receiving respective treatment, they were incubated for 48 h at 37 ℃ with 5% CO_2_. As instructed in β-Galactosidase detection kit, we sucked out the cell culture medium, and washed cells with PBS once. An appropriate amount of SA-β-Gal staining fixative for fixation was added into cells for 15 min at room temperature. Afterwards, the cell fixative was removed, and followed by 3 washes with PBS for 3 min each time. An appropriate amount of cell staining working solution was subsequently added into cells which were incubated overnight at 37 ℃. The aging cells were stained light blue to dark blue under the optical microscope.

### Flow cytometry detection

RGCs in logarithmic growth stage were inoculated into 6-well plates (2 ml/well) at a density of 4 × 10^8^ pieces/L. After culturing for 48 h, trypsin was added into each well for digestion. Being centrifuged at 1000 r / min for 5 min, cell precipitates were collected and rinsed twice with PBS. Later, according to the instructions of FITC-Annexin V apoptosis detection cell kit, flow cytometry was used to detect the apoptosis at the excitation wavelength of 488 nm and the apoptosis rate was calculated as: apoptosis rate = early apoptosis rate + late apoptosis rate [21].

The cells treated according to the above-method steps until twice washes with PBS, were supplemented with 75% ethanol for fixation overnight at 4 ℃. Subsequently, they were washed twice with 4 ℃ PBS and then centrifuged for 5 min at 1000 R / min. Propidium iodide [5] staining reagent was prepared according to the instructions of cell cycle kit, followed by a warm bath in dark at 37 ℃. Cell cycles were analyzed by flow cytometry [22]. The test was repeated 3 times.

### Gene sequencing

Gene sequencing was employed to elucidate variation of DEGs among normal, aging and USCs medium treated groups, 3 replicated wells for each group. The detailed steps were similar to our previously published article [23]. Then, the volcano plot analysis, enrichment analysis of Gene Ontology (GO) and Kyoto Encyclopedia of Genes and Genomes (KEGG) were performed for the biological function determination of DEGs.

### The Protein–Protein Interactions (PPIs) Network Analysis of Disease Intersection Targets

The PPIs network of disease targets was established and analyzed by STRING database (https://www.string-db.org/) [24]. The top 100 upregulated genes were imported into String for analysis. Those PPIs with high confidence score (> 0.90) should be selected for network construction and analysis to ensure the accuracy of the results. All the network can be constructed by employing the network visualization software Cytoscape 3.7.2 (https://cytoscape.org/). The connected genes from PPI network were imported into Cytoscpe for analysis of hub genes.

### GO and KEGG pathway enrichment analysis

GO and KEGG analysis was employed on all DEGs. GO enrichment analysis provides all GO terms that are significantly enriched in targets compared to the genome background and filters the targets that correspond to biological functions [25]. Pathway-based analysis is employed to characterize the biological functions of targets [26]. KEGG pathway enrichment analysis identified significantly signal transduction pathways in targets. In the present study, top 100 upregulated genes were mapped into Metascape database (http://metascape.org/gp/index.html#/main/step1), and gene numbers were calculated for every term.

### Statistical analysis

Graphpad prism 8.0 software was used for statistical analysis. Data were expressed as mean ± standard deviation (SD), and one-way analysis of variance (ANOVA) was used for comparison among more than 3 groups. *P* value less than 0.05 is considered statistically significant.

## Results

### Effects of D-galactose on survival rate of RGC cells

**Fig. 1:**
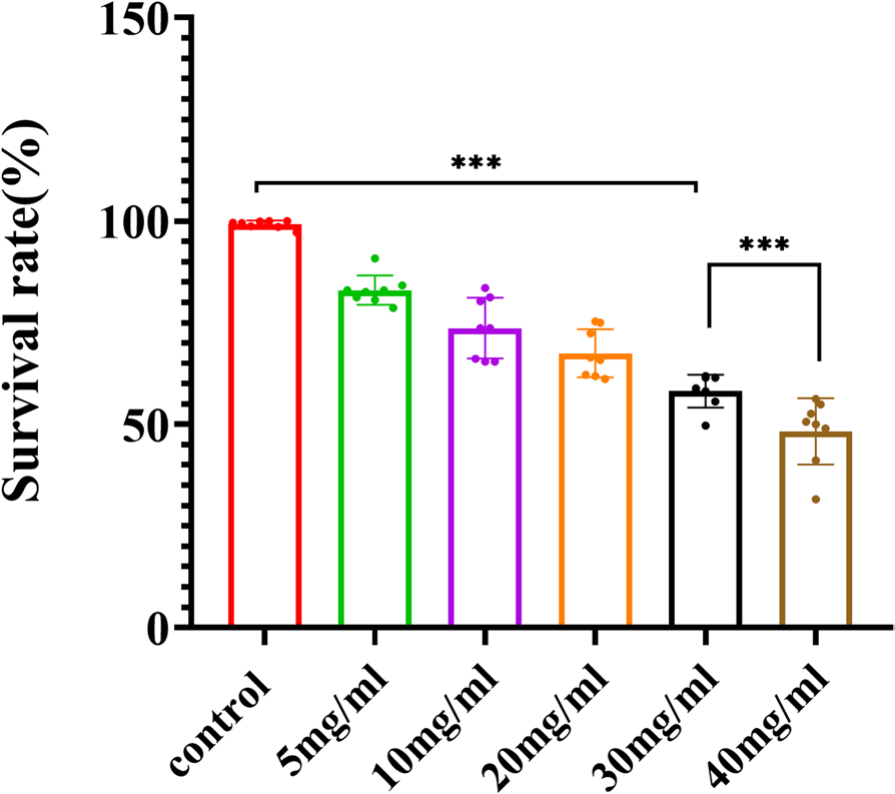
Survival rate of RGCs treated with different concentrations of D-galactose. *N* = 8 /group, ***p < 0.001

The cell viability of RGCs treated with different concentrations of D-galactose was detected by CCK8 assay. As demonstrated in Table 1, the cell survival rate was negatively correlated with the concentrations of D-galactose. With the concentration of D-galactose increasing, the survival rate gradually decreased, reaching about 58% when treated with 30 mg/mL D-galactose. The survival rate at 30 mg/mL was significantly lower than that of control group (*p* < 0.001, Fig. 1). Thus, 30 mg/mL D-galactose was determined to be the final concentration of inducing aging RGCs.

**Table 1:** Survival rate of RGCs treated with different concentrations of D-galactose

Groups	Concentrations	Survival rate
Control group		100%
Aging group	5 mg/ml	83% ± 0.017
	10 mg/ml	73.68% ± 0.036
	20 mg/ml	67.52% ± 0.02
	30 mg/ml	58.17% ± 0.0196
	40 mg/ml	48.27% ± 0.028

### Identification of USCs

**Fig. 2:**
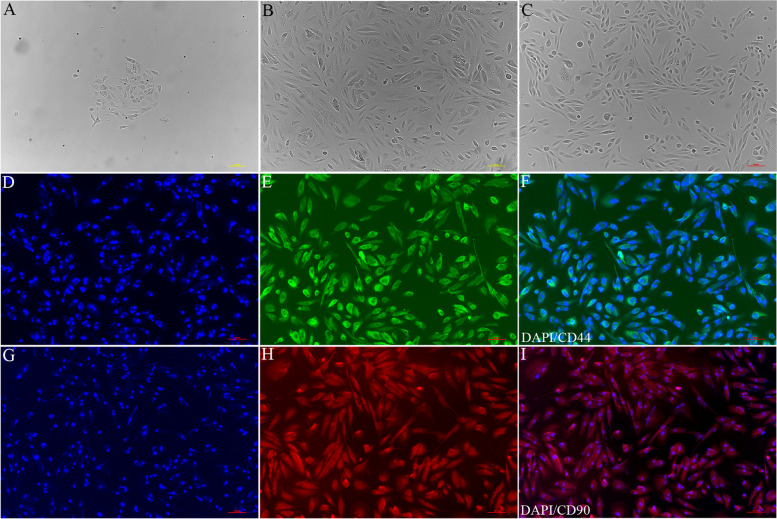
Immunostaining of CD44 and CD90 for USCs identification. Green fluorescence indicates CD44 positive cells, and red fluorescence indicates CD90 positive cells. Scale bar = 100 μm, *n* = 6 /group

Immunostaining results demonstrated that over 99% positive expression of CD44 and CD90 identified the purity of cultured USCs (Fig. 2). The high percentage of cells expressing CD44 and CD90 indicates MSCs characteristics of USCs.

### Apoptosis of aging RGCs was inhibited after USCs conditioned medium treatment

**Fig. 3:**
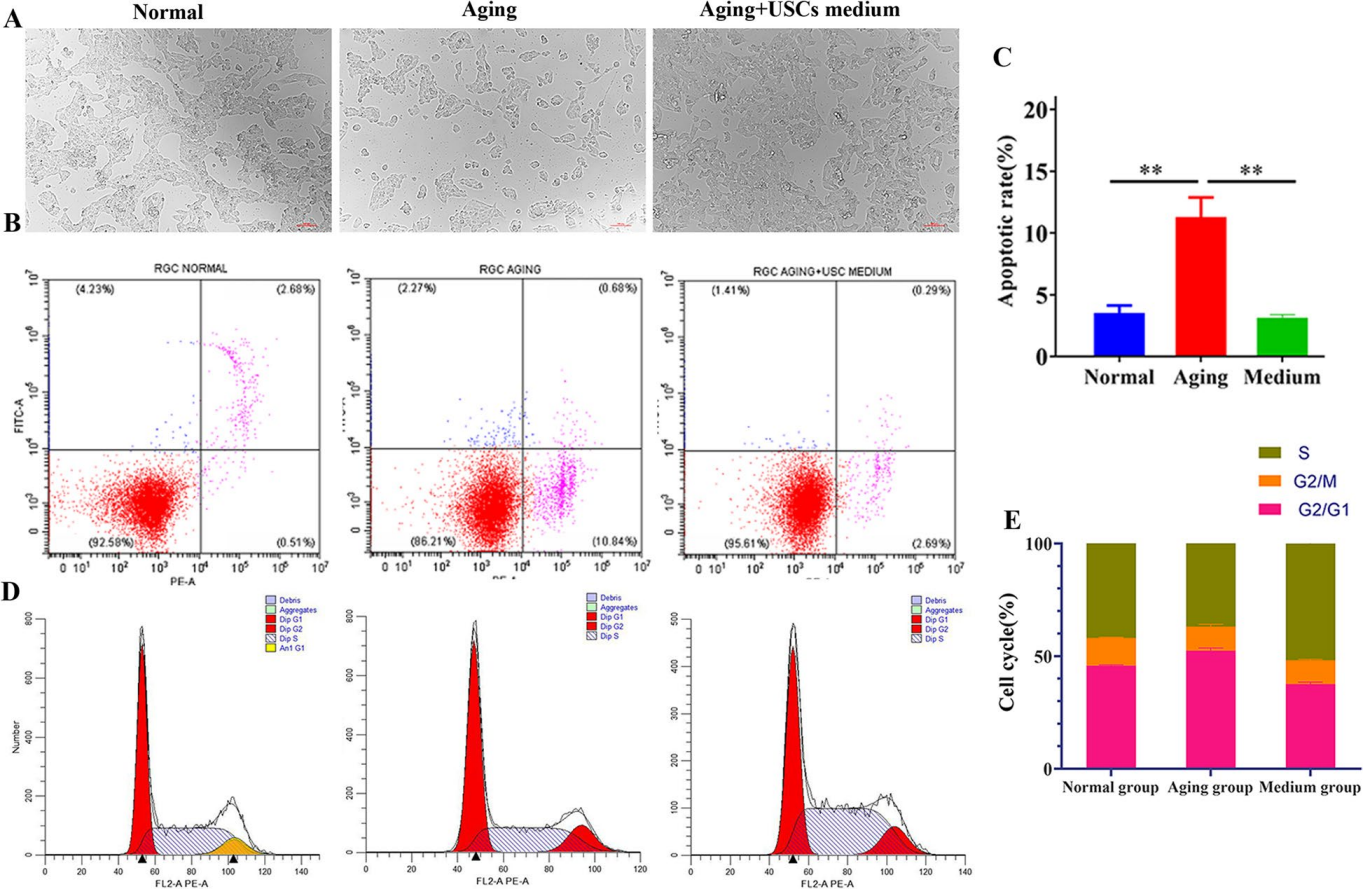
Apoptosis and cell cycle of RGCs among normal, aging and USCs medium groups. **A** Light field images of RGCs in normal group, aging group and aging + USCs medium group, scale bar = 100 μm. **B** The flow cytometry detection and **C** the apoptosis rate of RGCs among normal, aging and aging + USCs medium groups. **D** The cell cycle and **E** cycle rate of RGCs among normal, aging and aging + USCs medium groups. All data are shown as the mean ± SD, *n* = 6 /group. **p < 0.01

Flow cytometry was employed to detect the apoptosis rate of RGCs. The results revealed that apoptotic RGCs were significantly increased in the aging group, while the number of apoptotic cells got smaller in the aging RGCs cultured in USCs medium (Fig. 3A, B). Correspondingly, the apoptosis rate in aging group was much higher than that of normal group (*p* < 0.01, Fig. 3C), but it was markedly diminished in RGCs treated with USCs medium (*p* < 0.01, Fig. 3C). The results revealed that USCs-derived exosomes could inhibit the apoptosis of aging RGCs. Further, the cell count of RGCs in aging group at Freq G1 was largely increased, but obviously decreased at Freq S compared with that of the normal group (Fig. 3D, E). No obvious variation was revealed in the number of cells at Freq G2 among normal, aging and USCs medium groups (Fig. 3D, E). Relative to normal group, there’s a prominent increase of the cell number observed at Freq G1 in aging group, while a significant reduction at Freq G1 in USCs medium group (Fig. 3D, E).

### USCs conditioned medium promoted survival and proliferation of aging RGCs

**Fig. 4:**
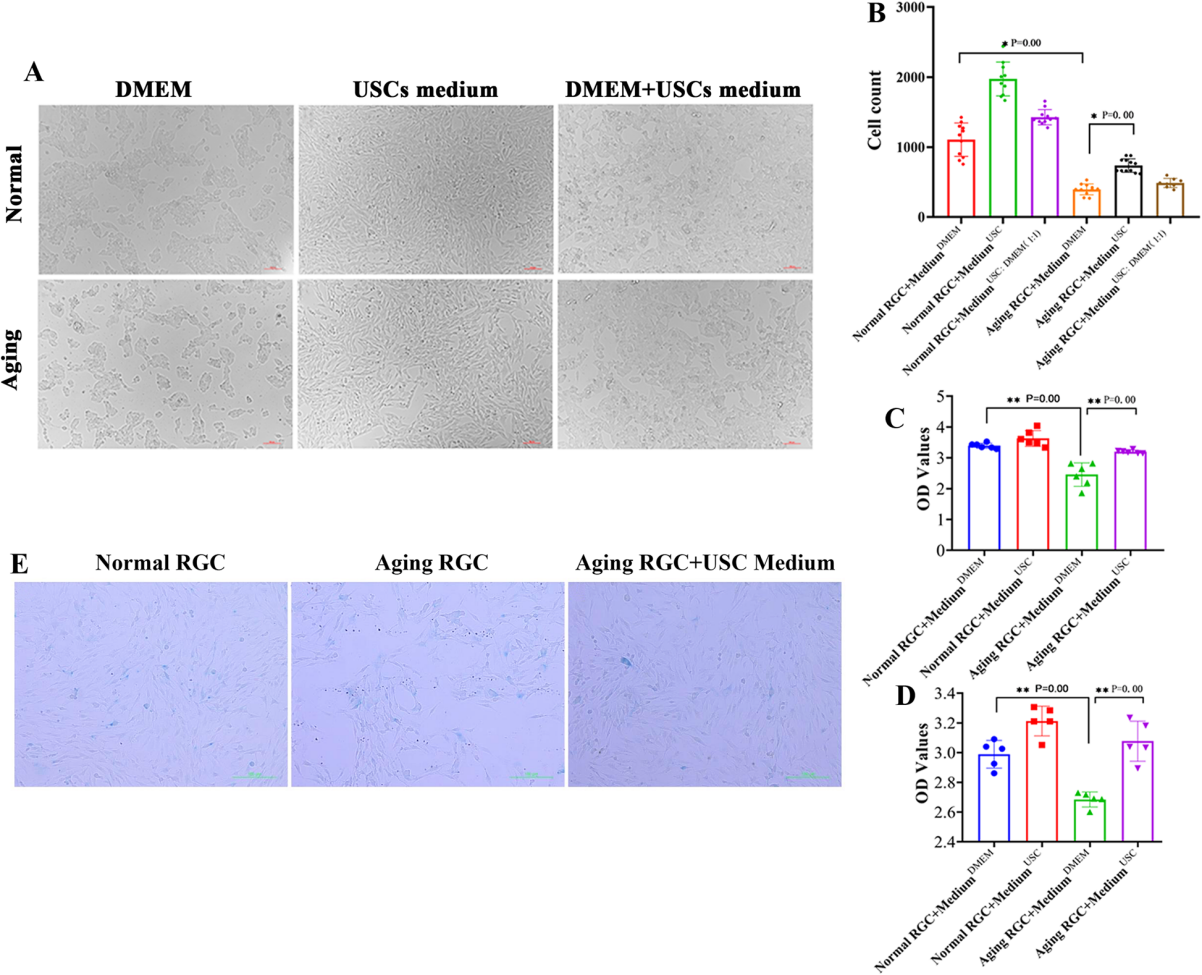
The effect of USCs conditioned medium on ameliorating aging RGCs. **A** Light field images of RGCs in normal and aging groups cultured in three kinds of culture medium, respectively, scale bar = 100 μm. **B** The cell number of RGCs in three kinds of culture medium between normal and aging groups, *n* = 12/group. OD value of RGCs in normal + DMEM medium, normal + USCs medium, aging + DMEM medium and aging + USCs medium groups at (**C**) 24 h and (**D**) 48 h, *n* = 6/group. **E** β-Galactosidase staining images of RGCs in normal + DMEM medium, aging + DMEM medium and aging + USCs medium groups, scale bar = 100 μm, *n* = 6/group

Three kinds of medium (DMEM; USCs conditioned medium; DMEM to USCs conditioned medium at 1:1) were used to culture RGCs in normal and aging groups. Normal RGCs in DMEM are irregular hexagon-shaped with uniform size, growing in good state (Fig. 4A). When cultured in USCs conditioned medium, the number of normal RGCs was increased and arranged more closely. In the combined medium with DMEM and USCs conditioned medium, variation in cell morphology was observed and cell number was reduced (Fig. 4A). When aging RGCs were cultured in DMEM, the cell count was significantly decreased relative to that of normal RGCs in DMEM (*p* = 0.00, Fig. 4B). However, aging cells in USCs conditioned medium were augmented markedly compared to cells in DMEM medium (*p* = 0.00, Fig. 4A, B) and combined medium. Cell viability was detected using CCK8 test at 24 h and 48 h, which demonstrated that in DMEM medium, the OD value of aging RGCs significantly reduced compared to that of normal RGCs at 24 h and 48 h (*p* = 0.00), while aging RGCs in USCs conditioned medium showed obvious augmented OD value in comparison to aging RGCs in DMEM at 24 h and 48 h (*p* = 0.00, Fig. 4C, D). The results of β-Galactosidase staining uncovered that there were more aging cells in DMEM medium, decreased number of aging RGCs and increased number of normal cells when cultured in USCs conditioned medium (Fig. 4E).

### Identification of DEGs and analysis on their biological functions

**Fig. 5:**
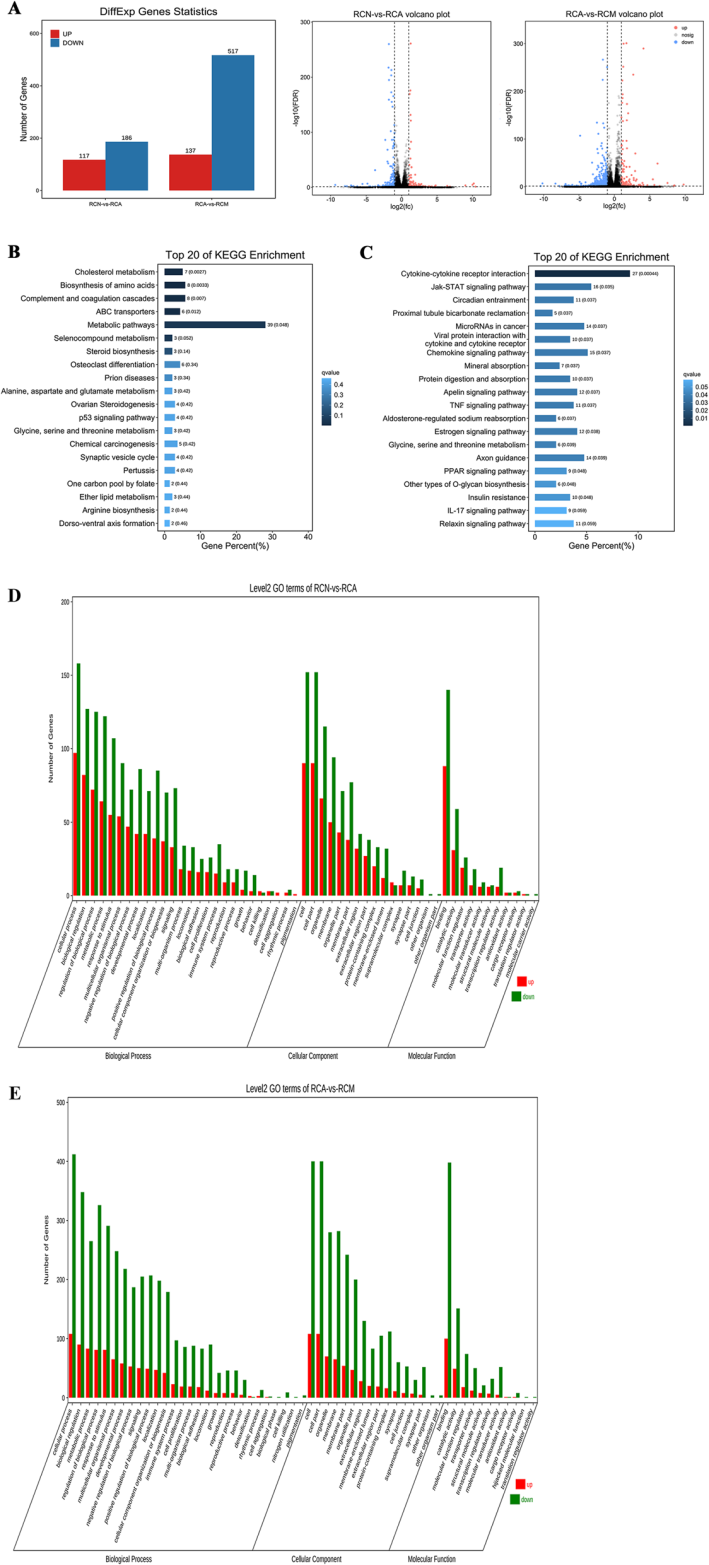
Bioinformatics analysis of DEGs involved in the effect of USCs-derived exosomes on aging RGCs. **A** The number of DEGs and volcano maps in normal RGCs group vs aging RGCs group, and aging RGCs group vs aging RGCs + USCs medium group, *n* = 3/group. **B**, **C** The enriched KEGG pathways involved by DEGs. **D**, **E** GO terms enriched by DEGs

In order to further investigate the underlying mechanisms related to the effect of USCs-derived exosomes on aging RGCs, the gene sequencing was performed to analyze the potential genetic variation. The sequencing outcomes demonstrated 117 upregulated genes and 186 downregulated genes in normal RGCs group vs aging RGCs group, 137 upregulated ones and 517 downregulated ones in aging RGCs group vs aging RGCs + USCs medium group (Fig. 5A). These DEGs underwent subsequent GO and KEGG analysis. As revealed, the top 20 enriched KEGG pathways involved by DEGs in aging RGCs vs normal RGCs were presented: Cholesterol metabolism Biosynthesis of amino acids, Complement and coagulation cascades, ABC transporters Metabolic pathways, Seleno compound metabolism, Steroid biosynthesis Osteoclast differentiation, Prion diseases, Alanine, aspartate and glutamate metabolism, Ovarian Steroidogenesis p53 signaling pathway, Glycine, Serine and threonine metabolism, Chemical carcinogenesis, Synaptic vesicle cycle, Pertussis, One carbon pool by folate, Ether lipid metabolism Arginine biosynthesis, Dorso-ventral axis formation (Fig. 5B). Meanwhile, the top 20 enriched KEGG pathways related to DEGs in aging RGCs vs aging cells cultured in USCs conditioned medium were Cytokine-cytokine receptor interaction (map04060), Jak-STAT signaling pathway (map04630), Circadian entrainment (map04713), Proximal tubule bicarbonate reclamation (map04964), MicroRNAs in cancer (map05206), Viral protein interaction with cytokine and cytokine receptor (map04061), Chemokine signaling pathway (map04062), Mineral absorption (map04978), Protein digestion and absorption (map04974), Apelin signaling pathway (map04371), TNF signaling pathway (map04668), Aldosterone-regulated sodium reabsorption (map04960), Estrogen signaling pathway (map04915), Glycine, serine and threonine metabolism (map00260), Axon guidance (map04360), PPAR signaling pathway (map03320), Other types of O-glycan biosynthesis (map00514), Insulin resistance (map04931), IL-17 signaling pathway (map04657), Relaxin signaling pathway (map04926). In addition, the GO terms DEGs involved are also displayed (Fig. 5C). Top 10 biological processes in which DEGs in aging RGCs vs normal cells participated included cellular process, biological regulation, regulation of biological process, metabolic process, response to stimulus, multicellular organismal process, negative regulation of biological process, developmental process, localization, positive regulation of biological process (Fig. 5D). Cell components of these DEGs include cell, cell part, organelle, membrane, organelle part, membrane part, extracellular region. Molecular functions of these DEGs involved mainly include binding, catalytic activity, molecular function regulator, transporter activity (Fig. 5D). Comparatively, top 10 biological processes in which DEGs in aging cells vs aging RGCs + USCs medium participated involved cellular process, biological regulation, metabolic process, regulation of biological process, response to stimulus, multicellular organismal process, developmental process, negative regulation of biological process, signaling, positive regulation of biological process (Fig. 5E). Cell components of these DEGs included cell, cell part, organelle, membrane, membrane part, organelle part, extracellular region, membrane-enclosed lumen. Molecular functions of these DEGs involved binding, catalytic activity, molecular function regulator, transporter activity (Fig. 5E).

## Discussion

Vision loss resulting from damage and death of RGCs is usually irreversible as human get older and older, which causes extremely miserable life experience to those suffering vision disorders. MSCs are advantageous cells in regenerative medicine, exerting pleiotropic functions by producing soluble factors, such as exosomes [17]. Here, we established aging RGCs models and carried out cultures of aging RGCs in USCs conditioned medium, aiming to reveal the potential efficacy of USCs-derived exosomes in reversing the aging process of RGCs and boost functional recovery of aging RGCs. The results demonstrated positive therapeutic effect of USCs-derived exosomes on the cell viability and proliferation of RGCs. Further, sequencing data identified differential genes expressed in aging RGCs and aging RGCs treated with USCs medium and analyzed their biological functions.

Damage or death of RGCs is usually accompanied by functional alterations. A previous study reported that functional alterations to RGCs in inner retinal neurons already appear before the following detected evidence of cell degeneration [27]. Besides, early signs of damage have been detected at the level of RGCs’ axons [28, 29], subsequently, RGCs cell bodies shrink and their dendrites are pruned before cell death [6, 30. Thus, it’s necessary for us to focus on the morphologies of RGCs prior to the detection of RGCs functions since defects in RGCs function appear later than its variation in morphology. In this study, we applied morphological detection and observed the morphological variation and cell count change of aging RGCs. Cell bodies of aging RGCs exhibited obvious shrinkage and pruned dendrites, along with sharp reduction of cell number. Comparatively, the morphology and amount aging cells cultured in USCs conditioned medium nearly approached the RGCs at normal level. CCK8 assay and flow cytometry detection further proved the cell viability of aging RGCs was enhanced in USCs conditioned medium, and the number of apoptotic aging RGCs was also significantly reduced, which suggested that USCs-derived exosomes might have the capabilities of inhibiting apoptosis of aging RGCs and help recover the cell viability of aging RGCs.

Being derived from the convoluted tubules of nephron, renal pelvis, ureters, bladder and urethra, USCs have a similar phenotype to MSCs and can be reprogrammed into induced pluripotent stem cells [31]. The therapeutic potential of USCs in urologic surgeries and dysfunctions has been shown in previous publications [13]. USCs were induced to differentiate into urothelial and smooth muscle cells in vitro and then seeded on small intestinal submucosa scaffolds which were then implanted into nude mice. Then, multilayered tissue-like structures composed of urothelium and smooth muscle were visualized, which proved the mighty feasibility to apply USCs as a source of cells for tissues-engineered strategies [31]. In addition, it has been demonstrated the abilities of USCs differentiating into neurogenic cells. After transplantation of USCs into rat brains, they survived in the lesion site, migrated to other brain areas, and expressed neurogenic markers such as GFAP, β-III tubulin and nestin [32]. These outcomes revealed the abilities of USCs differentiating into neuron-like cells in the rat brain and their possibilities for regenerative medicine, which correspondingly provide evidence for the efficacy of USCs in rescuing the viability of aging RGCs. Strikingly, in addition to MSCs themselves, their exosomes are capable of mediating intercellular communication, targeting cells inducing cellular development through different signaling pathways. Exosomes produced by endothelial cells exhibit distinct cargoes that may be useful as a biomarker of age-related vascular disease [33]. Also, MSCs-derived exosomes can improve ageing and age-related diseases [17]. Consistently, our work revealed that exosomes from USCs-derived MSCs inhibited the apoptosis of aging RGCs and enhanced the cell viability of aging RGCs. Consequently, USCs-derived exosomes might present a potential therapeutic method for RGCs loss-induced vision disorders.

Nevertheless, the detailed mechanisms underlying the efficacy of USCs-derived exosomes in hampering age-related disorders remain yet elusive. It’s critical to inform the understanding of molecular characterization of aging RGCs and specify the stereotyped connections underlying their physiological function. Therefore, we’ve analyzed the genetic variation of normal RGCs vs aging RGCs and aging RGCs vs aging RGCs with USCs conditioned medium by employing gene sequencing. Our sequencing data displayed 117 upregulated genes and 186 downregulated genes in normal RGCs group vs aging RGCs group, 137 upregulated ones and 517 downregulated ones in aging RGCs group vs aging RGCs + USCs medium group, suggesting the aging RGCs and the effect of USCs-derived exosomes involved massive genetic variation inside. It has been reported that early signs of damage have been detected at the level of RGCs’ axons involving metabolic changes, impairment of axonal transport, and downregulation of specific genes [28, 29]. Similarly, the 5 primary biological processes in which DEGs in aging RGCs and aging RGCs in USCs medium participated in are Cellular process, Biological regulation, Regulation of biological process, Metabolic process, Response to stimulus. Among the enriched KEGG pathways involved by DEGs from aging RGCs, the top five are Cholesterol metabolism Biosynthesis of amino acids, Complement and coagulation cascades, ABC transporters metabolic pathways, Selenocompound metabolism, Steroid biosynthesis Osteoclast differentiation. Comparatively, of the enriched KEGG pathways involved by DEGs from aging RGCs in USCs medium, the top 10 included Cytokine-cytokine receptor interaction, JAK-STAT signaling pathway, Circadian entrainment, Proximal tubule bicarbonate reclamation, MicroRNAs in cancer Viral, protein interaction with cytokine and cytokine receptor Chemokine signaling pathway, Mineral absorption, Protein digestion and absorption, Apelin signaling pathway TNF signaling pathway, Aldosterone-regulated sodium reabsorption. JAK/STAT signaling pathway is known as a universally expressed intracellular signal transduction pathway and took active action in many crucial biological processes, including cell proliferation, differentiation, apoptosis, and immune regulation [34]. These revealed that the effect of USCs-derived exosomes activates many positive molecular activities to promote the recovery of RGCs function. A separate study suggested an induction in the exosomal secretory pathway coincided with a block in autophagy progress, accelerating senescence, which might be targeted for the treatment of age-related diseases [35]. Targeting exosomes represents novel approaches as cell-free therapy and drug-delivery system for the treatment of age-related diseases. Although exosomes-therapy may serve as a new approach to combat ageing, the translation of preclinical results to clinic needs more extensive investigation on exosomes both on their biology and related techniques. Overall, scrutiny on the effect of ageing on USCs and vice versa is vital for designing novel therapy using USCs with focus on the management of older individuals.

## Conclusions

On the whole, the therapeutic potentials of USCs-derived exosomes for aging RGCs were presented in this study, including suppression on cell apoptosis, enhancement on cell viability and proliferation of RGCs. The underlying mechanism involves multiple genetic variation and changes of transduction signaling pathways, which provide referential basis for the later in-depth mechanistic investigations on the efficacy of USCs-derived exosomes in the age-related disease.

## Data Availability

The raw sequence data reported in this paper have been deposited in the Gene Expression Ominibus of National Center for Biotechnology Information, under accession number GSE224109 and are publicly accessible at https://www.ncbi.nlm.nih.gov/geo/query/acc.cgi?acc=GSE224109. The token number (edszuwosnzevxit) is provided for review only.
